# Supplementary material for: EnzML: multi-label prediction of enzyme classes using InterPro signatures
Source: BMC Bioinformatics. 2012 Apr 25;13:61. doi: 10.1186/1471-2105-13-61 (PMC3483700; doi:10.1186/1471-2105-13-61)
Supplement: Addtional file 5 — The Java code to format the data files, evaluate and predict. The file enzml_java_code.tar.gz contains the Java code used to format database data to ARFF and XML formats, to execute cross and train-test (jackknife) evaluations and to record evaluation results to database. More information is included in the readme.txt file and the Javadoc files. The code can be used with a MySQL database. To use a different database software, other JDBC drivers might be required. [file 1471-2105-13-61-S5.gz › java_code/enzml2011/doc/uk/ac/ed/inf/enzml/mulan/attributesfilter/AttributesFilter.html]

AttributesFilter


---


|  |  |  |
| --- | --- | --- |
| **Overview** \| **Package** \| **Class** \| **Use** \| **Tree** \| **Deprecated** \| **Index** \| **Help** | |  |
| PREV CLASS   **NEXT CLASS** | **FRAMES**    **NO FRAMES**     **All Classes** |  |
| SUMMARY: NESTED \| FIELD \| CONSTR \| METHOD | DETAIL: FIELD \| CONSTR \| METHOD |  |


---


## uk.ac.ed.inf.enzml.mulan.attributesfilter Class AttributesFilter

```
java.lang.Object
  uk.ac.ed.inf.utils.Initialised
      uk.ac.ed.inf.enzml.mulan.attributesfilter.AttributesFilter
```

**All Implemented Interfaces:**: uk.ac.ed.inf.utils.Initialisable

---

``` public class AttributesFilter extends uk.ac.ed.inf.utils.Initialised ```

This class filters the attributes of a dataset (the test set) to make it
compatible with another dataset (the train set). The result is a new dataset
that can be used as test set for the specified train set.
Main operations: 1. Attributes: only attributes present in the train set can
appear in the test set (& all of them must be present) 2. Class attributes:
all the classes of the train set \*and\* the test set must be conserved, to
allow for train/test evaluation. This means that also the train arff must be
regenerated to include all the classes present in the test set (the class
numbering will change). 3. Mulan XML file (class labels): cannot be the same
XML file of the training set, must also include all the classes in the test
set. 4. New test ARFF File: the new test set arff file name should mention
the original files and which attributes and classes were used for filtering
(that is, left in the file or added to the file). 5. New train ARFF file:
(including also the test classes) 'trainset id' + 'including\_classes\_of' +
'test\_set id' + '.arff' 6. New test ARFF record: when stored in the database,
should contain trace (id/props file path) of the original training set and
the "old" test set used to generate the "new", filtered, test set.

**Version:**
:   3 Sep 2010

**Author:**
:   Luna De Ferrari luna.deferrari-at-ed.ac.uk

---

**Field Summary**

**Fields inherited from class uk.ac.ed.inf.utils.Initialised**

`m_initialisationIsCorrect`


**Constructor Summary**

`AttributesFilter(int oldTrainArffId,
int oldTestArffId,
java.lang.String dataDatabaseProps,
java.lang.String arffAndResultsPropsFileName)`


| **Method Summary** | |
| --- | --- |
| `int` | `createNewArff(int oldArffRecordId)` |
| `void` | `createNewTestArff()` |
| `void` | `createNewTrainArff()` |
| `java.lang.String` | `getArffAndResultsProperties()` |
| `uk.ac.ed.inf.utils.database.DbManager` | `getArffRecordsDbManager()` |
| `ArffPropsTableReader` | `getArffTableReader()` |
| `uk.ac.ed.inf.utils.database.DbManager` | `getDataDbManager()` |
| `java.lang.String` | `getDataDbPath()` |
| `int` | `getNewTestArffId()` |
| `int` | `getNewTrainArffId()` |
| `int` | `getOldTestArffId()` |
| `int` | `getOldTrainArffId()`             public MulanArff getTrainArff() { return m\_trainArff; } |
| `java.util.TreeSet<java.lang.String>` | `getTrainAttributesAndTrainTestClasses()` |
| `java.lang.String` | `getTrainXmlPath()` |
| `java.lang.String` | `getXmlPathById(int arffId)` |
| `java.lang.String` | `getXmlQueryById(int arffId)` |
| `static void` | `main(java.lang.String[] args)` |
| `static int[]` | `run(int oldTrainArffId, int oldTestArffId, java.lang.String dataDbPropsFile, java.lang.String resultsPropsFileName)`             Filter the old test arff by the train arff given and regenerate the old train set using all train+test classes. |

**Methods inherited from class uk.ac.ed.inf.utils.Initialised**

`initialisationIsCorrect, setInitialisationIsCorrect`

**Methods inherited from class java.lang.Object**

`equals, getClass, hashCode, notify, notifyAll, toString, wait, wait, wait`

**Constructor Detail**

### AttributesFilter

```
public AttributesFilter(int oldTrainArffId,
                        int oldTestArffId,
                        java.lang.String dataDatabaseProps,
                        java.lang.String arffAndResultsPropsFileName)
```


**Method Detail**

### createNewArff

```
public int createNewArff(int oldArffRecordId)
```

:   **Parameters:**: `oldArffRecordId` - the database id of the old arff file **Returns:**: the database id of the new, filtered arff file

---


### createNewTestArff

```
public void createNewTestArff()
```

---


### createNewTrainArff

```
public void createNewTrainArff()
```

---


### getArffAndResultsProperties

```
public java.lang.String getArffAndResultsProperties()
```

---


### getArffRecordsDbManager

```
public uk.ac.ed.inf.utils.database.DbManager getArffRecordsDbManager()
```

---


### getArffTableReader

```
public ArffPropsTableReader getArffTableReader()
```

---


### getDataDbManager

```
public uk.ac.ed.inf.utils.database.DbManager getDataDbManager()
```

---


### getDataDbPath

```
public java.lang.String getDataDbPath()
```

---


### getNewTestArffId

```
public int getNewTestArffId()
```

---


### getNewTrainArffId

```
public int getNewTrainArffId()
```

---


### getOldTestArffId

```
public int getOldTestArffId()
```

---


### getOldTrainArffId

```
public int getOldTrainArffId()
```

:   public MulanArff getTrainArff() { return m\_trainArff; }

---


### getTrainAttributesAndTrainTestClasses

```
public java.util.TreeSet<java.lang.String> getTrainAttributesAndTrainTestClasses()
```

---


### getTrainXmlPath

```
public java.lang.String getTrainXmlPath()
```

---


### getXmlPathById

```
public java.lang.String getXmlPathById(int arffId)
```

---


### getXmlQueryById

```
public java.lang.String getXmlQueryById(int arffId)
```

---


### main

```
public static void main(java.lang.String[] args)
```

---


### run

```
public static int[] run(int oldTrainArffId,
                        int oldTestArffId,
                        java.lang.String dataDbPropsFile,
                        java.lang.String resultsPropsFileName)
```

:   Filter the old test arff by the train arff given and regenerate the old
    train set using all train+test classes.

    :   **Parameters:**: `oldTrainArffId` - the record id of the original training arff: `oldTestArffId` - the record id of the original test arff: `resultsPropsFileName` - the database where to write the new arff records: `dataDbPropsFile` - the database where to read the data to rebuild the arff files **Returns:**: the identifiers of the new train and test arff records in the database


---


|  |  |  |
| --- | --- | --- |
| **Overview** \| **Package** \| **Class** \| **Use** \| **Tree** \| **Deprecated** \| **Index** \| **Help** | |  |
| PREV CLASS   **NEXT CLASS** | **FRAMES**    **NO FRAMES**     **All Classes** |  |
| SUMMARY: NESTED \| FIELD \| CONSTR \| METHOD | DETAIL: FIELD \| CONSTR \| METHOD |  |


---
